# Intergenerational transmission of attachment: The role of intelligence

**DOI:** 10.1002/jcv2.70013

**Published:** 2025-04-25

**Authors:** Jana Runze, Marinus H. Van IJzendoorn, Annemieke M. Witte, Charlotte A. M. Cecil, Marian J. Bakermans‐Kranenburg

**Affiliations:** ^1^ Research Institute of Child Development and Education University of Amsterdam Amsterdam the Netherlands; ^2^ Research Department of Clinical, Education and Health Psychology Faculty of Brain Sciences UCL London UK; ^3^ Clinical Child & Family Studies Faculty of Behavioral and Movement Sciences Vrije Universiteit Amsterdam the Netherlands; ^4^ Department of Child and Adolescent Psychiatry and Psychology Erasmus MC University Medical Center Rotterdam Rotterdam the Netherlands; ^5^ William James Center for Research University Institute of Psychological, Social and Life Sciences ISPA Lisbon Portugal; ^6^ Facultad de Psicología y Humanidades Universidad San Sebastián Valdivia Chile; ^7^ Department of Psychology Stockholm University Stockholm Sweden

**Keywords:** IQ, polygenic scores, secure base script, sensitivity

## Abstract

**Background:**

In their recent paper, Del Giudice and Haltigan argue that attachment in childhood and attachment representations in adulthood are influenced by the cognitive capabilities of children and parents, that would causally link parents' attachment states of mind to children's attachment.

In the current pre‐registered study, we empirically explored the idea of an association between attachment and cognition using phenotypic child IQ and parent and child IQ‐related polygenic scores as predictors of children's attachment behavior and attachment representations.

**Methods:**

We used data from the Leiden Consortium on Individual Development study (L‐CID, *n* = 992), a two‐cohort longitudinal twin study, in which attachment representations were measured in parents and their 9‐year‐old children using the Attachment Script Assessment (ASA). Polygenic scores of IQ were computed for parents and their children using PRSice‐2 and phenotypic child IQ was measured as well. We split the twin sample in two groups randomly to prevent non‐independence of data and conducted structural equation models.

**Results:**

Neither parental nor child polygenic scores of IQ predicted representations of attachment. In one cohort, phenotypically measured IQ predicted attachment.

**Conclusions:**

This preliminary study did not find convincing support for a role of IQ in the intergenerational transmission of attachment.

## Graphical abstract

Attachment and cognitive development are associated, but the direction of the association is unclear. Recently, Del Giudice and Haltigan have argued that attachment in childhood and attachment representations in adulthood are influenced by the cognitive capabilities of the individual, which might make the intergenerational transmission of attachment spurious. We found that children's polygenic score of IQ was not associated with continuously measured attachment (representations) in a longitudinal cohort study.

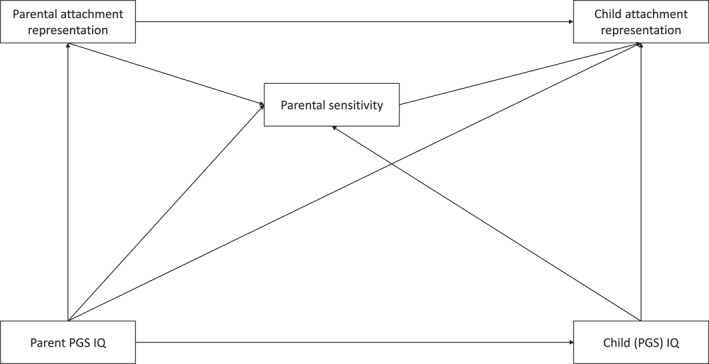

**Key points**

- Recently, Del Giudice and Haltigan (2023) have argued that attachment in childhood and attachment representations in adulthood are influenced by the cognitive capabilities of the individual, which might make the intergenerational transmission of attachment spurious.
- We found that children's polygenic scores of IQ were not associated with their attachment representations in a longitudinal two‐cohort study.
- We do not consider their model refuted by the current study but in need of further refinement and replicated testing in independent, powerful causal inference designs

## INTRODUCTION

In over 50 years of attachment research, the child‐parent attachment relationship has been linked to many developmental outcomes, such as internalizing behaviors (Madigan et al., 2013), externalizing behaviors (Fearon et al., 2010), temperament (Dagan et al., 2023; Groh et al., 2017a, 2017b), prosociality (Deneault, Hammond, & Madigan, 2023) and social competence (Groh et al., 2017a, 2017b). In a recent meta‐analysis including more than 6000 children, attachment was associated with cognitive and language development (Deneault, Duschinsky, et al., 2023). The results are usually interpreted as secure attachment stimulating positive development in the socio‐emotional and cognitive domains. However, the direction of results in these mostly correlational studies is unclear.

Therefore, in the current pre‐registered study, we tested a model of associations between attachment, parental sensitivity, and intelligence, as proposed by Del Giudice and Haltigan (2023). Their central argument is that child attachment as well as attachment representations in adulthood are influenced by intelligence, and that the genetic correlation between parent and child intelligence gives rise to a spurious pathway linking parents' attachment to children's attachment. Parental intelligence would exert an additional indirect influence on children's attachment through its association with parenting sensitivity (Bakermans‐Kranenburg, 2006). Based on this model, they suggest that measures of intelligence would help to (partly) bridge the “transmission gap” between parental attachment representations and child attachment, as shared cognitive resources would explain (some of) the association between parent and child attachment. In the current study, we empirically investigated the association between attachment and cognition using both phenotypic IQ and polygenic indicators of child and parent intelligence as predictors of their attachment representations, and of parental sensitivity.

Attachment theory suggests that children are born with the capacity to form an attachment relationship with their caregivers and that children display attachment behaviors to survive and receive support and comfort in times of stress and distress (Bowlby, 1982). The type of attachment relationships that children develop partly depends on the caregivers' sensitive parenting behavior (van IJzendoorn et al., 2023; Verhage et al., 2016). Parents who are sensitive tend to have securely attached children, whereas parents who are harsh, inconsistent or rejecting tend to have insecurely attached children (Chang et al., 2003; De Wolff & van IJzendoorn, 1997; Verhage et al., 2016).

Attachment has been speculated to predict not only socio‐emotional development but also cognitive and language development (van IJzendoorn et al., 1995). Four hypotheses were proposed to explain this association. The first suggests that in secure dyads, children's cognitive resources are not used for coping with attachment‐related stress, so they can concentrate on processing cognitive input from their caregiver. The second posits that secure children are more at ease in new environments, and therefore more relaxed exploring them which might foster cognitive development. The third proposes that children with a secure attachment are more socially competent and profit more from interactions with others. The fourth suggests that securely attached children have better cooperation skills in stressful testing situations, resulting in higher test scores for cognition and language. The radical reversal of the association, that is, that cognitive and language development determine the quality of the attachment relationship, is mentioned but not elaborated by van IJzendoorn et al. (1995). The most recent meta‐analysis, including 76 studies with 6831 children, showed that attachment security measured in infancy or toddlerhood was positively associated with cognitive development in 3‐year‐olds, *r* = 0.17 (Deneault, Duschinsky, et al., 2023). The authors interpreted this predictive association in a unidirectional way such that a secure attachment relationship may predict better cognition, but they left room for reversed causality.

**FIGURE 1:**
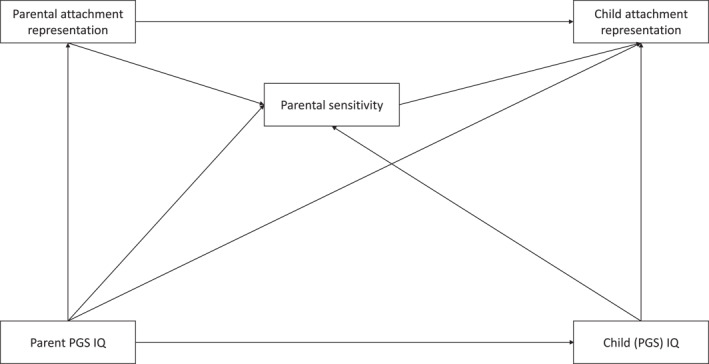
Primary hypotheses of the current study based on the model proposed by Del Giudice and Haltigan (2023). For simplicity, direct effects arrows are omitted.

Following from their hypothesis that cognitive capabilities affect attachment quality, Del Giudice and Haltigan (2023) speculate that parental IQ might play a role in the intergenerational transmission of attachment. The intergenerational transmission of attachment refers to associations between parental attachment and child attachment. Intergenerational transmission is partly but not fully explained by parental sensitivity, and the unexplained transmission is referred to as the transmission gap (van IJzendoorn, 1995; Verhage et al., 2016). According to Del Giudice and Haltigan (2023), parental intelligence might (partly) close this gap, as it may predict parental attachment, sensitivity and mentalizing, partial mediators between parental attachment and attachment of the child. This perspective thus introduces an alternative pathway through which intelligence may contribute to the transmission of attachment across generations (see Figure 1). Their model assumes that parental intelligence influences parental attachment representation and sensitive parenting, and child intelligence has an impact on child attachment (see Del Giudice & Haltigan, 2023). The specific role of intelligence in intergenerational transmission of attachment has not been thoroughly investigated yet.

In this pre‐registered study (https://doi.org/10.17605/OSF.IO/ETB47), we investigated whether cognition predicts children's and parent's attachment representations. More specifically, we examined the potentially causal influence of IQ on attachment security. We chose IQ as a proxy for the more general domain of cognitive development. In addition to phenotypical IQ scores, we used a polygenic score of cognition (PGS‐IQ). The advantage of using a PGS is that it is based on inherited DNA and cannot be influenced by attachment. At the same time, measuring PGS‐IQ still is developing and only a proxy for phenotypical intelligence, explaining up to 7% of the variance in phenotypically measured intelligence (Allegrini et al., 2019; Genç et al., 2021, 2023; Loughnan et al., 2023). Although this percentage seems modest, in the area of molecular genetics of complex cognitive and behavioral traits it is one of the stronger indicators. We used data from the L‐CID study, a two‐cohort twin study with an early childhood cohort (ECC) (*M*_*age*_ at assessment of attachment = 9 years) and a middle childhood cohort (MCC), (*M*_*age*_ at assessment of attachment = 10 years) in which attachment representations were measured in parents and their children using the Attachment Script Assessment (ASA, Waters & Waters, 2006, 2021).

We tested whether phenotypical child IQ and child and parent PGS‐IQ would predict attachment representations in parents and children. In accordance with replicated models of intergenerational transmission of attachment (van IJzendoorn, 1995; Verhage et al., 2016), we expected that the association between parental attachment representations and child attachment would be mediated by parental sensitivity. To examine Del Giudice and Haltigan's (2023) model, we also tested whether the association between parental and child attachment was mediated by parental sensitivity when controlling for phenotypic or polygenic IQ (see Figure 1).

## MATERIALS AND METHODS

### Participants

Participants took part in the L‐CID study, an experimental accelerated longitudinal twin study with two cohorts: an ECC (*n* = 239 families) and a MCC (*n* = 257 families). Families with same‐sex twins from the western region of the Netherlands were contacted through municipality records and invited for participation if the twins had the same gender, their parents were Dutch speaking and parents as well as grandparents were European. Six yearly assessments (T1–T6) were conducted with alternating home or lab visits. An intervention (VIPP‐SD; Juffer et al., 2017) was implemented between T2 and T3. Intervention effects are reported elsewhere (Euser, et al., 2021; Runze, Van IJzendoorn, et al., 2022) and controlled for in the current study. For a detailed description of the recruitment, inclusion and exclusion criteria see Euser et al. (2021) and van der Meulen et al. (2018) for the ECC and MCC, respectively. At T1, the children in the ECC were on average 3.79 years old (SD = 0.58) and children in the MCC 7.95 years old (SD = 0.67). At the time of the attachment assessment, the children in the ECC were on average 9.09 years old (*SD* = 0.61, T6) and in the MCC 9.98 years old (*SD* = 0.69, T3). 60% of the children were monozygotic twins and 45% were male. 91% and 92% of the participating parents self‐identified as primary caregivers in the ECC and MCC respectively were mothers.

### Measures

#### Parental attachment representations

At T2, we measured parent's secure base script knowledge (SBSK‐P) with the ASA (Waters & Waters, 2006, 2021). The ASA comprises six stories, two neutral stories and four attachment stories. To reduce the burden on participants, we used four stories: one neutral story and three attachment stories, a practice reliably employed in previous studies (Cuyvers et al., 2023; Verhees et al., 2021; Waters et al., 2015, 2019). Parents produced four different stories based on 12‐word prompt sets with a title. The prompt words indicated a possible story with a beginning, middle, and end. The neutral prompt word was used for practice purposes. Two other word sets implied hypothetical mother‐child relationships (Baby's Morning and Doctor's Office) and one word set concerned a hypothetical adult–adult relationship (The Accident). Stories were scored on a seven‐point rating scale (1 = poorly developed secure base script to 7 = well‐developed secure base script). All stories were double coded by reliable coders and scores of both coders were averaged. In case of deviations of more than one point, a consensus score was assigned through discussion. Coders were blind to which story belonged to which parent. The mean ICC with the expert coder [AMW] was 0.78 based on 120 stories. Cronbach's alpha for the three stories was *α* = 0.65. Scores of the three stories were averaged into one score indicating parent's secure base script knowledge (SBSK‐P).

#### Child attachment representations

Children's secure base script knowledge (SBSK‐C) was assessed with the Middle Childhood ASA (Waters et al., 2019). Children were asked to tell four stories based on prompt word sets with a title and 12 prompt words. Of the four stories, one was developed to elicit a neutral story and three were attachment related stories, named “Scary dog in the yard”, “At the beach” and “Field hockey game”. This third title was changed from the original “Soccer game” because in the Netherlands field hockey is commonly played by both girls and boys, whereas soccer is still less popular among girls. Of note, no changes were made to the prompt words. Each story was coded by two coders and scores assigned by the two coders were averaged. When scores deviated more than one point, a consensus score was reached through discussion. The mean ICC with expert coder [AMW] was 0.86. Scores of the three narratives were averaged into an overall score of SBSK‐C. Internal consistency was high, *α* = 0.81.

#### Observed parental sensitivity

Sensitivity of the primary parent was observed at T3 using a computerized version of the Etch‐A‐Sketch task (Cents et al., 2014). The parent performed the task with each twin separately, in random order. The parent‐child dyad copied three printed drawings with increasing difficulty on a computer screen. Sensitivity was coded from video recordings using the revised (Egeland, 1990) seven‐point rating scales for Supportive Presence (1 = parent completely fails to be supportive to the child, 7 = parent skillfully provides support throughout the session) and Intrusiveness (1 = parent allows the child sufficient time to explore and to attempt to solve tools on her/his own, 7 = parent is highly intrusive; her/his agenda clearly has precedence over the child's wishes, reverse coded). A mean score was computed as scores on the two scales correlated substantially (*r* = 0.57). The mean ICC with the expert coder was adequate (ICC = 0.74) (see Euser et al., 2021; Runze et al., 2022a; Runze et al., 2022b).

#### IQ

Children of the MCC completed the third edition of the WISC‐III (Wechsler, 1991) at T1. The WISC‐III is an intelligence test for 6‐ to 16‐year‐old children, with three composite IQ scores, full‐scale IQ, verbal IQ and performance IQ. In the ECC, children completed the verbal IQ scale of the WPPSI (Wechsler, 1967) at T3 and the performance IQ scale at T4. The WPPSI is designed for 2‐ to 7‐year‐old children and provides three composite IQ scores, full‐scale IQ, verbal IQ and performance IQ. We used the full‐scale IQ scores in our analyses. Phenotypic IQ was not assessed in parents.

#### Polygenic scores (PGS)

Saliva samples were collected from parents and their children. DNA was genotyped by the genetics laboratory of the Department of Internal Medicine (Population Genomics) at Erasmus MC using the GSA‐MD array (version 2). Quality control was performed in PLINK and using zCall software. We removed SNPs with low call rate (< 97.5%), low minor allele frequency (<5%) or if they were not in Hardy‐Weinberg equilibrium (HWE, *p* < 1*10^−5^). Genotypes were imputed using the 1000 Genomes Project (phase III release version 5), applying a two‐step imputation and using GRCh37/hg19 build as reference panel, see Runze, van IJzendoorn, et al. (2022), for a detailed description of the analysis. We used the GWAS summary statistics of Savage et al. (2018). To compute the polygenic score of IQ, we used the PRSice‐2 software (Choi & O’Reilly, 2019) wherein the GWAS summary statistics served as a base sample and L‐CID was the target sample. We only used autosomal SNPs as there is no consensus for sex chromosomes (Choi & O’Reilly, 2019). The PGS were calculated using clump *r* ^*2*^ = 0.1, 250 kb at different *p*‐value thresholds (i.e., 1, 0.50, 0.40, 0.30, 0.20, 0.10, 0.05, and 0.001). We tested the PGS using linear regression models and selected the *p*‐value threshold of the PGS with the largest *R* ^2^ for further analyses (see Table S2a for *p*‐value thresholds). Selecting the best *p‐*value threshold is comparable to tuning parameter optimization (Choi & O’Reilly, 2019) and the risk of overfitting is minimal (Krapohl et al., 2018). To account for differences in ancestry, we residualized the PGS by regressing out the effects of five principal components (Abegaz et al., 2019), and conducted subsequent analyses using the residualized PGS.

### Data analysis

First, we tested whether possible covariates (age, sex, intervention status, socio‐economic status) were significantly associated with the outcome variables. Then, we assessed missingness using Little's MCAR test (naniar package, Tierney & Cook, 2020) We used multiple imputation (MI) to impute missing data (mice package, Van Buuren & Groothuis‐Oudshoorn, 2011) with 20 iterations for each of the 48 imputed datasets. Multiple imputation is preferable above other techniques of handling missing data and has a reduced risk of biased estimates (Madley‐Dowd et al., 2019). Data points that deviated ≥ 3.29 *SD* from the mean were winsorized (parental PGS‐IQ = 0, Child PGS‐IQ = 2, SBSK‐P = 12, SBSK‐C = 10, parental sensitivity = 0, Child IQ = 2, Table S2b). We computed the ICC to assess whether the SBSK‐C within families was more similar than across families. Because of a rather high ICC (0.29), we split the twin pairs randomly into two samples and conducted the analyses in both samples separately. We employed structural equation models (lavaan package, Rosseel, 2012) using full information maximum likelihood and Yuan‐Bentler scaled Chi‐square estimator with Huber‐White covariance adjustment to the standard errors. We assessed model fit using the Comparative Fit Index (CFI; Bentler, 1990), the Tucker–Lewis Index (TLI; Tucker & Lewis, 1973), and Root Mean Square Error of Approximation (RMSEA; Hu & Bentler, 1998). Good model fit is assumed with CFI and TLI values greater than 0.95 and RMSEA smaller than 0.08 (Xia & Yang, 2019). A priori power was computed with the shiny app MedPower (MedPower; Kenny, 2017). Power for all paths ranged from 0.10 to 0.99, depending on assumed effects (between 0.10 and 0.17), and sample size (175 as smallest sample size). Equivalence testing using TOSTER (Lakens, 2017) was conducted in cases of null results. We deviated from the pre‐registration (https://doi.org/10.17605/OSF.IO/ETB47), wherein we wanted to replicate our study in a second sample. However, in this sample, child attachment and IQ were not significantly correlated, the data therefore did not hold true for the premise of our study question. For transparency reasons we reported the deviation from the pre‐registration on OSF at 2024‐12‐17 including an explanation why the results for the Generation R study (originally intended to be included) were left out of the current report (see: https://doi.org/10.17605/OSF.IO/ETB47).

## RESULTS

### Descriptive analyses

Parental and child PGS‐IQ were positively correlated, although somewhat lower than expected based on familial relationship (*r* = 0.39). SBSK‐C was positively correlated with child IQ (*r* = 0.15) but not with child PGS‐IQ (*r* = 0.06). Child PGS‐IQ and phenotypic IQ were positively correlated (*r* = 0.21). See Table 1 for all descriptive statistics. Missingness occurred not completely at random (X^2^ (323) = 1314.48, *p* < 0.001, see also Figure S6 and Table S1 for detailed information on drop‐out and missingness) and we imputed data using MI as suggested in the literature (e.g., Newman, 2014). Girls had higher SBSK‐C scores than boy, and children of older parents and of parents from higher SES had higher SBSK‐C scores compared to children of younger parents and parents from lower SES. Parents who participated in the intervention, parents from higher SES and mothers (compared to fathers) had higher SBSK‐P scores. Parents from higher SES and parents in the intervention group were more sensitive. These covariates were therefore included in subsequent analyses (Table S3).

**TABLE 1:** Means, standard deviations, and correlations.

Variable	*N*	*M*	*SD*	1	2	3	4	5	6	7
1. Parental age at T1	980	38.42	5.02							
2. Child age^a^	674	9.59	0.79	**0.36**						
3. Child IQ	875	103.30	11.35	0.00	0.02					
4. Parental PGS IQ^b^	586	−0.00	0.00	**0.15**	−0.03	**0.16**				
5. Child PGS IQ^b^	372	−0.00	0.00	**0.17**	−0.05	**0.21**	**0.39**			
6. Parental sensitivity	843	4.00	1.34	−0.05	−0.01	**0.13**	0.08	0.09		
7. SBSK‐P	906	3.96	0.66	0.06	0.05	**0.10**	**0.10**	0.06	**0**.**15**	
8. SBSK‐C	737	3.27	0.39	**0.11**	**0.23**	**0.15**	0.05	**0.14**	0.03	0.02

*Note*: *M* and *SD* are used to represent mean and standard deviation, respectively; Significant correlations (*p* < 0.05) are shown in bold.

Abbreviations: PGS, polygenic score; SBSK‐C, secure base script knowledge of the child; SBSK‐P, secure base script knowledge of the parent.

^a^ Child age at assessment of attachment representations.

^b^ Since the PGS is based on a large GWAS, the beta coefficients per SNP are very small, resulting in a PGS with a mean close to 0 and a small but nonzero SD.

### Main analyses

We employed structural equation models to examine whether parental or child polygenic scores of IQ would predict SBSK‐C. None of the variables emerged as significant predictor, neither for the first, nor for the second child (see Table 2). There was no significant mediation effect in either of the two subsamples: The potential transmission of attachment representations was not mediated by parental sensitivity. Likewise, parental sensitivity did not mediate an association between parental PGS‐IQ and SBSK‐C.

**TABLE 2:** Structural equation model of parental and child PGS IQ, parental sensitivity, and phenotypic IQ on the child attachment representations.

	Child 1						Child 2					
Predictors	b	se	z	*p*	CI		b	se	z	*p*	CI	
SBSK‐C	*R* ^2^ = 6.4%, Cohen's *d* = 0.52						*R* ^2^ = 7.7%, Cohen's *d* = 0.58					
Parent PGS‐IQ	−0.03	0.06	−0.53	0.60	−0.15	0.09	0.03	0.06	0.50	0.62	−0.08	0.14
Child PGS‐IQ	0.02	0.05	0.33	0.74	−0.09	0.12	0.02	0.06	0.44	0.66	−0.09	0.13
Child IQ	0.10	0.05	1.94	0.05	0.00	0.21	0.09	0.05	1.70	0.09	−0.01	0.19
SBSK‐P	0.01	0.05	0.18	0.86	−0.10	0.12	0.01	0.05	0.22	0.83	−0.09	0.11
Sensitivity (b1)	−0.02	0.06	−0.38	0.71	−0.13	0.09	0.04	0.05	0.83	0.41	−0.06	0.14
Parent age	0.02	0.01	1.80	0.07	0.00	0.04	0.01	0.01	1.24	0.22	−0.01	0.03
Child sex	**0.39**	**0.11**	**3.60**	**0.00**	**0.18**	**0.60**	**0.46**	**0.10**	**4.45**	**0.00**	**0.26**	**0.66**
SES	0.07	0.09	0.71	0.48	−0.11	0.24	0.07	0.09	0.78	0.44	−0.10	0.24
Sensitivity	R^2^ = 7.5%, Cohen's *d* = 0.57						R^2^ = 3.6%, Cohen's *d* = 0.39					
Parent PGS‐IQ (a1)	−0.03	0.06	−0.46	0.64	−0.14	0.08	0.04	0.06	0.75	0.45	−0.07	0.16
Child PGS‐IQ (a2)	0.04	0.05	0.79	0.43	−0.06	0.14	0.00	0.06	0.00	1.00	−0.11	0.12
SBSK‐P (a3)	0.09	0.05	1.66	0.10	−0.02	0.19	0.09	0.06	1.59	0.11	−0.02	0.20
SES	**0.36**	**0.08**	**4.34**	**0.00**	**0.20**	**0.52**	**0.19**	**0.09**	**2.18**	**0.03**	**0.02**	**0.37**
Intervention group	0.13	0.10	1.21	0.23	−0.08	0.33	0.14	0.11	1.29	0.20	−0.08	0.36
SBSK‐P	R^2^ = 4.4%, Cohen's *d* = 0.43						R^2^ = 4.5%, Cohen's *d* = 0.43					
Parent PGS‐IQ	0.02	0.06	0.28	0.78	−0.10	0.13	0.04	0.06	0.80	0.43	−0.07	0.15
Parent sex	0.36	0.19	1.85	0.06	−0.02	0.74	0.37	0.19	1.92	0.06	−0.01	0.74
SES	**0.28**	**0.09**	**3.20**	**0.00**	**0.11**	**0.44**	**0.26**	**0.09**	**3.04**	**0.00**	**0.09**	**0.43**
Intervention group	0.15	0.11	1.34	0.18	−0.07	0.36	0.13	0.11	1.24	0.22	−0.08	0.35
Child PGS‐IQ	R^2^ = 5.6%, Cohen's *d* = 0.49						R^2^ = 3.4%, Cohen's *d* = 0.38					
Parent PGS‐IQ	**0.25**	**0.06**	**4.42**	**0.00**	**0.14**	**0.36**	**0.18**	**0.05**	**3.57**	**0.00**	**0.08**	**0.28**
Mediation												
a1*b1	0.00	0.00	0.25	0.80	−0.01	0.01	0.00	0.00	0.45	0.65	−0.01	0.01
a2*b1	0.00	0.01	−0.18	0.86	−0.01	0.01	0.00	0.00	0.03	0.98	0.00	0.00
a3*b1	0.00	0.01	−0.40	0.69	−0.01	0.01	0.00	0.01	0.59	0.55	−0.01	0.02

*Note*: b = unstandardized parameter estimate, Model fit: Child 1: Χ^2^ (34) = 84.47, *p* = 0.001, CFI = 0.99, TLI = 0.98, RMSEA = 0.008, Child 2: Χ^2^ (34) = 69.31, *p* = 0.001, CFI = 0.93, TLI = 0.86, RMSEA = 0.017; model fit was poor for child 2, which can be expected in models with many variables and a modest sample size, and we therefore did not try to improve model fit using modification indices (Kenny & McCoach, 2003); Significant correlations (*p* < 0.05) are shown in bold.

Abbreviations: CI, confidence interval; SBSK‐C, secure base script knowledge of the child; SBSK‐P, secure base script knowledge of the parent; se, standard error; *z*, Z‐statistic.

### Sensitivity analyses

As pre‐registered, we repeated the structural equation modeling using the latent variable PGS‐EDINQ (i.e., a latent factor comprised of polygenic scores of IQ, education and income, as done in Runze et al. [2023]) and using complete data (*n* = 197) instead of the imputed data. The results were similar, but in the complete data analysis, observed child IQ predicted child SBSK‐C scores (Child 1: *β* = 0.18, *p* = 0.01, Child 2: *β* = 0.16, *p* = 0.02, Tables S4 and S5). We also repeated the main analyses omitting the path between parental PGS‐IQ and SBSK‐P to test whether a potential collider bias might influence the results, but results were similar to the main model (Table S6). Moreover, we repeated the main analyses without SES as covariate (but including child sex and age) to assess potential overcontrolling via SES, but results were similar to the main model as well (Table S7). Lastly, we also repeated the main analyses but using polygenic scores with a *p*‐value threshold of 1, and results were the same (Table S8).

### Equivalence testing

Equivalence testing using the TOSTER package (Lakens, 2017) revealed that the non‐significant association between child attachment and parental PGS was practically equivalent to zero (*p*'s > 0.05 given alpha = 0.05 and equivalence bounds of −0.99 and 0.10, in accordance with recommendations of Schuengel et al. (2021)), whereas the non‐significant association between child PGS‐IQ and attachment (SBSK‐C) was not equivalent to zero (*p* = 0.03 respectively, *α* = 0.05, equivalence bounds of −0.99 and 0.10).

## DISCUSSION

In the current pre‐registered study, we tested a model of associations between attachment, parental sensitivity, and intelligence, as proposed by Del Giudice and Haltigan (2023). Their central argument is that child attachment as well as attachment states of mind in adulthood are influenced by intelligence, and that the genetic correlation between parent and child intelligence gives rise to a spurious pathway linking parents' attachment to children's attachment. Parental intelligence would exert an additional indirect influence on children's attachment through its association with parenting sensitivity. Based on this model, they suggest that measures of intelligence would help to (partly) bridge the “transmission gap” between parental state of mind and child attachment, as shared cognitive resources would explain the overlap in parent and child attachment.

Importantly, because it is virtually impossible to reliably measure infants' cognitive capabilities before they develop attachment relationships, the directionality of an association between attachment and cognitive development is difficult to establish when relying on phenotypic IQ measures. A stringent test of Del Giudice and Haltigan's (2023) model thus includes polygenic scores of IQ (PGS‐IQ), that cannot be influenced by attachment. This is what we did in the current study, relating phenotypic IQ and PGS‐IQ to attachment and sensitivity measures, using a cohort study including twins which offers a great opportunity for internal replication.

### Intelligence and attachment representations in middle childhood and adults

We examined the potential association between intelligence and attachment representations at an age for which Del Giudice and Haltigan (2023) hypothesized that the association between intelligence and (representational measures of) attachment would be stronger than the association between intelligence and (behavioral) attachment measures in infancy. The PGS‐IQ did not explain variance in attachment representations, neither in the parents nor in the children. Moreover, no associations between phenotypical IQ and attachment representations were found. The absence of associations between PGS‐IQ and attachment representations in both twin cohorts strengthens the assumption that there is no (unidirectional) causal pathway from IQ to attachment representations. Equivalence testing showed that the non‐significant association between parental PGS‐IQ and child attachment representations was practically zero, but not for the non‐significant association between child PGS‐IQ and child attachment representations.

### Intergenerational transmission of attachment

Del Giudice and Haltigan (2023) argue that the “correlation between parent and child intelligence gives rise to an indirect causal pathway linking parent and child attachment… (parent state of mind ← parent intelligence ↔ child intelligence → child attachment). This pathway should increase the intergenerational concordance [of attachment] between parents and children without the mediation of parents' caregiving behavior—thus inflating the apparent size of the transmission gap.” (p. 13). We agree with the implication of a potentially spurious association between parental and child attachment inflating the estimated intergenerational attachment transmission. If parent and child intelligence indeed were related to attachment, this indirect path should be discounted resulting in a smaller or even non‐existent transmission gap.

Yet, in the current study the assumptions of Del Giudice and Haltigan's (2023) argument were not confirmed. First, we did not find robust evidence for a unidirectional influence of intelligence on parental or child attachment representations. We found an association between phenotypically measured IQ and attachment representations in sensitivity analyses using complete data only, but in none of the other main or sensitivity analyses. Second, PGS‐IQ did not predict attachment representations in the parents. For a (partially) spurious association both variables should be (partly) predicted by the ‘confounder’, which was not the case. Furthermore, we did not find a causal influence of child PGS‐IQ on observed parental sensitivity, although we agree with Del Giudice and Haltigan (2023) that some type of active gene‐environment correlation (Knafo & Jaffee, 2013) might be expected.

Our study has some limitations that preclude a final verdict on the transmission model that Del Giudice and Haltigan (2023) proposed. We used data from only one parent. Including both parents would allow us to further quantify any presence of (in‐)direct genetic effects, as well as relative contributions of mothers versus fathers in the transmission of attachment. Further work is also clearly needed with adolescent samples and other attachment representation assessments such as the widely validated Adult Attachment Interview (Main et al., 1985). The concept of attachment should not be confused with its measures and a multimethod approach with attachment assessments on various levels of functioning will prove to be indispensable in answering complex questions and models such as Del Giudice and Haltigan proposed. The same might be true for the age‐old concept of intelligence. Going beyond the traditional way of measuring IQ phenotypes, we included polygenic scores for IQ that have the advantage of unequivocal causal unidirectionality to child characteristics. Of course, measuring PGS‐IQ still is developing and only a proxy for phenotypical intelligence. Although it is currently one of the most powerful polygenic scores in psychology and psychiatry, it explains only part of the variance in IQ, so a lack of evidence of associations with the PGS‐IQ does not preclude residual associations not yet explained by the PGS. Despite current limitations of polygenic scores we emphasize the important role of exploratory studies to show new directions (e.g., addressing the problem of reversed causality in attachment research with PGSs) and to serve as input for (individual participant data) meta‐analytic combinations of a series of relatively small studies in research areas that are necessarily dependent on resource‐intensive assessments (van IJzendoorn & Bakermans‐Kranenburg, 2024).

## CONCLUSION

Del Giudice and Haltigan (2023) should be commended for re‐opening the discussion about the role of intelligence in attachment measures beyond infancy, including (largely verbal) assessments of cognitive representations or schemas in children, adolescents, and adults. It addresses early psychometric concerns about the Adult Attachment Interview (Bakermans‐Kranenburg & van IJzendoorn, 1993) and the SBSK (Bakermans‐Kranenburg, 2006). Del Giudice and Haltigan also correctly point out that a substantial causal impact of intelligence on parental and child attachment might imply narrowing of the intergenerational attachment transmission gap. Our current study was triggered by their ‘bold conjecture’ of an alternative model that seemed plausible and, most importantly, testable with measures that were not available a decade ago. The current limitations of polygenic scores, however, substantially reduce the power of our analyses and point at the need for much larger samples. We do not consider their model refuted by the current study but in need of further refinement and replicated testing in independent, more powerful designs with causal implications.

## AUTHOR CONTRIBUTIONS

**Jana Runze:** Conceptualization; formal analysis; investigation; methodology; visualization; writing—original draft; writing–review and editing. **Marinus H. Van IJzendoorn:** Conceptualization; funding acquisition; methodology; supervision; writing—original draft; writing—review and editing. **Annemieke M. Witte:** Data curation; investigation; writing—original draft. **Charlotte A. M. Cecil:** Project administration; resources; writing—review and editing. **Marian J. Bakermans‐Kranenburg:** Conceptualization; funding acquisition; methodology; supervision; writing—original draft; writing—review and editing.

## CONFLICT OF INTEREST STATEMENT

The authors declare no conflicts of interest.

## ETHICAL CONSIDERATIONS

Ethical approval for the L‐CID study was provided by the central committee on research involving human subjects (CCMO; NL49069.000.14). All participants provided written informed consent.

## Supporting information

Supplementary Material

## Data Availability

The data that support the findings of this study are available upon request (see www.developmentmatters.nl/data‐access/).
