# Supplementary material for: Intergenerational transmission of attachment: The role of intelligence
Source: JCPP Adv. 2025 Apr 25;5(4):e70013. doi: 10.1002/jcv2.70013 (PMC12698279; doi:10.1002/jcv2.70013)
Supplement: Supplementary file 1 — Supplementary Material [file JCV2-5-e70013-s001.docx]

| **Supporting Information** | | |  |  |  |  |  |
| --- | --- | --- | --- | --- | --- | --- | --- |
|  |  |  |  |  |  |  |  |
| **Intergenerational Transmission of Attachment: The Role of Intelligence** | | | | | | | |
| **Running title:** Intelligence and transmission of attachment | | | | | |  |  |

| **Figure S1** |  |  |  |
| --- | --- | --- | --- |
|  |  |  |  |
| *Participation and drop-out of both cohorts of the L-CID study* | | | |

| **Table S1** |  |  |  |  |  |  |  |
| --- | --- | --- | --- | --- | --- | --- | --- |
|  |  |  |  |  |  |  |  |
| *Differences between participating and non-participating families in both cohorts* | | | | |  |  |  |
|  |  |  |  |  |  |  |  |
|  | Families participating at T6 | | Families not participating at T6 | |  |  |  |
|  |  |  |  |  |  |  |  |
|  | *N* | % | *N* | % | χ^2^ | *p* |  |
| Sex child (boys) | 351/386 | 47.63 | 129/120 | 51.57 | 1.14 | 0.29 |  |
| Sex parent (Fathers) | 68/669 | 9.58 | 14/239 | 5.62 | 2.91 | 0.09 |  |
| SES (low) | 44/307/384 | 5.99 | 32/107/112 | 12.75 | **13.3** | **<.01** |  |
|  |  |  |  |  |  |  |  |
|  | *M* | *SD* | *M* | *SD* | *t* | *p* |  |
| Age child | 6.14 | 2.14 | 5.37 | 2.16 | **4.89** | **<.01** |  |
| Age parent | 38.68 | 5.09 | 37.64 | 4.74 | **2.94** | **<.01** |  |
| IQ child | 103.72 | 11.24 | 101.53 | 11.66 | **2.21** | **0.03** |  |
| *Note.* Significant results are depicted in bold; | | | | | | |  |

| **Table S2a** |  |  |  |
| --- | --- | --- | --- |
|  |  |  |  |
| *PGS thresholds for the continuous attachment measure* | | | |
|  |  |  |  |
| PGS | Threshold | Parent | Child |
| PGS IQ | 0.001 | **0.010480** | 0.002583 |
| PGS IQ | 0.05 | 0.006638 | 0.011770 |
| PGS IQ | 0.1 | 0.004078 | 0.010726 |
| PGS IQ | 0.2 | 0.003417 | 0.015388 |
| PGS IQ | 0.3 | 0.001857 | 0.016985 |
| PGS IQ | 0.4 | 0.002814 | 0.017884 |
| PGS IQ | 0.5 | 0.002717 | **0.018575** |
| PGS IQ | 1 | 0.002678 | 0.014781 |
| PGS EA | 0.001 | 0.004898 | 0.001196 |
| PGS EA | 0.05 | 0.001146 | 0.003109 |
| PGS EA | 0.1 | 0.002019 | 0.003091 |
| PGS EA | 0.2 | 0.003249 | 0.002117 |
| PGS EA | 0.3 | 0.004237 | 0.003372 |
| PGS EA | 0.4 | 0.005012 | 0.003444 |
| PGS EA | 0.5 | **0.005220** | 0.003357 |
| PGS EA | 1 | 0.004561 | **0.004014** |
| PGS Income | 0.001 | 0.005118 | 0.001785 |
| PGS Income | 0.05 | 0.007747 | 0.005244 |
| PGS Income | 0.1 | 0.010942 | 0.007543 |
| PGS Income | 0.2 | 0.012728 | **0.011048** |
| PGS Income | 0.3 | 0.011267 | 0.004278 |
| PGS Income | 0.4 | 0.013974 | 0.004044 |
| PGS Income | 0.5 | **0.014206** | 0.004627 |
| PGS Income | 1 | 0.013251 | 0.004878 |
| *Note.* PGS = Polygenic score; EA = educational attainment. | | | |

| **Table S2b** |  |
| --- | --- |
|  |  |
| *Number of outliers* |  |
|  |  |
| Variable | Nr of outliers |
| Parent PGS EA | 4 |
| Parent PGS IQ | 0 |
| Parent PGS Income | 0 |
| Child PGS EA | 2 |
| Child PGS IQ | 2 |
| Child PGS Income | 0 |
| Parent SBSK | 12 |
| Child SBSK | 10 |
| Sensitivity | 0 |
| Child IQ | 2 |

| **Table S3** |  |  |
| --- | --- | --- |
|  |  |  |
| *Regression estimates to assess potential covariates* | | |

|  | Attachment Child (R² = 11.3%) | | | |  | Attachment Parent (R² = 4.3%) | | | |
| --- | --- | --- | --- | --- | --- | --- | --- | --- | --- |
| Predictor | *b* | *b* | *sr^2^* | *sr^2^* |  | *b* | *b* | *sr^2^* | *sr^2^* |
|  |  | 95% CI |  | 95% CI |  |  | 95% CI |  | 95% CI |
|  |  | [LL, UL] |  | [LL, UL] |  |  | [LL, UL] |  | [LL, UL] |
| (Intercept) | **2.79** | [2.50, 3.08] |  |  |  | **2.91** | [2.47, 3.36] |  |  |
| Age Parent | **0.05** | [0.03, 0.06] | 0.05 | [.02, .08] |  | 0 | [-0.02, 0.02] | 0 | [-.00, .00] |
| Age Child | 0 | [-0.01, 0.01] | 0 | [-.00, .00] |  | 0.01 | [-0.00, 0.02] | 0 | [-.00, .01] |
| Sex Parent | -0.04 | [-0.15, 0.06] | 0 | [-.00, .01] |  | **0.25** | [0.09, 0.41] | 0.01 | [-.00, .02] |
| Sex Child | **0.17** | [0.11, 0.22] | 0.05 | [.02, .07] |  | -0.04 | [-0.13, 0.05] | 0 | [-.00, .01] |
| SES | **0.06** | [0.01, 0.11] | 0.01 | [-.00, .02] |  | **0.16** | [0.09, 0.23] | 0.02 | [.00, .04] |
| Intervention group | 0.01 | [-0.04, 0.07] | 0 | [-.00, .00] |  | **0.11** | [0.02, 0.19] | 0.01 | [-.00, .02] |

*Note.* A significant *b*-weight indicates the semi-partial correlation is also significant. *b* represents unstandardized regression weights. *sr^2^* represents the semi-partial correlation squared. *LL* and *UL* indicate the lower and upper limits of a confidence interval, respectively.

| **Table S3 continued** |  |  |
| --- | --- | --- |
|  |  |  |
| *Regression estimates to assess potential covariates* | | |

|  |  | Sensitivity (R² = 4.7%) | | | |
| --- | --- | --- | --- | --- | --- |
| Predictor |  | *b* | *b* | *sr^2^* | *sr^2^* |
|  |  |  | 95% CI |  | 95% CI |
|  |  |  | [LL, UL] |  | [LL, UL] |
| (Intercept) |  | **3.35** | [2.41, 4.29] |  |  |
| Age Parent |  | -0.04 | [-0.09, 0.01] | 0 | [-.00, .01] |
| Age Child |  | -0.01 | [-0.03, 0.01] | 0 | [-.00, .01] |
| Sex Parent |  | 0.02 | [-0.31, 0.35] | 0 | [-.00, .00] |
| Sex Child |  | -0.03 | [-0.21, 0.15] | 0 | [-.00, .00] |
| SES |  | **0.39** | [0.24, 0.54] | 0.03 | [.01, .05] |
| Intervention group |  | **0.23** | [0.04, 0.41] | 0.01 | [-.00, .02] |

*Note.* A significant *b*-weight indicates the semi-partial correlation is also significant. *b* represents unstandardized regression weights. *sr^2^* represents the semi-partial correlation squared. *LL* and *UL* indicate the lower and upper limits of a confidence interval, respectively.

|  |  |  |  |  |  |  |  |  |  |  |  |
| --- | --- | --- | --- | --- | --- | --- | --- | --- | --- | --- | --- |
|  |  |  |  |  |  |  |  |  |  |  |  |

| **Table S4** |  |  |  |  |  |  |  |  |  |  |  |  |
| --- | --- | --- | --- | --- | --- | --- | --- | --- | --- | --- | --- | --- |
|  |  |  |  |  |  |  |  |  |  |  |  |  |
| *Structural equation model of parental and child PGS IQ, parental sensitivity, and phenotypic IQ on the child attachment representations using the latent variable PGS-EDINQ* | | | | | | | | | | | | |

|  | Child 1 | | | | | | Child 2 | | | | | |
| --- | --- | --- | --- | --- | --- | --- | --- | --- | --- | --- | --- | --- |
| Predictors | b | se | z | p | CI | | b | se | z | p | CI | |
| **Parent PGS-EDINQ** |  | |  |  |  |  |  |  |  |  |  |  |
| Parent PGS-EA | **1.00** |  |  |  |  |  | **1.00** |  |  |  |  |  |
| Parent PGS-Income | **0.75** | **0.16** | **4.83** | **0.00** | **0.44** | **1.05** | **0.91** | **0.19** | **4.81** | **0.00** | **0.53** | **1.28** |
| Parent PGS-IQ | **0.50** | **0.12** | **4.25** | **0.00** | **0.27** | **0.73** | **0.58** | **0.13** | **4.38** | **0.00** | **0.32** | **0.85** |
| **Child PGS-EDINQ** |  | |  |  |  |  |  |  |  |  |  |  |
| Child PGS-EA | **1.00** |  |  |  |  |  | **1.00** |  |  |  |  |  |
| Child PGS-Income | **0.82** | **0.10** | **7.97** | **0.00** | **0.62** | **1.03** | **1.09** | **0.18** | **6.10** | **0.00** | **0.74** | **1.44** |
| Child PGS-IQ | **0.31** | **0.07** | **4.34** | **0.00** | **0.17** | **0.46** | **0.26** | **0.08** | **3.21** | **0.00** | **0.10** | **0.42** |
| **Child Attachment** | **R² = 7.1%, Cohen’s d = 0.55** | | | |  |  | **R² = 7.4%, Cohen’s d = 0.57** | | | |  |  |
| Parent PGS-EDINQ | -0.03 | 0.12 | -0.27 | 0.79 | -0.28 | 0.21 | 0.01 | 0.13 | 0.05 | 0.96 | -0.25 | 0.26 |
| Child PGS-EDINQ | -0.03 | 0.08 | -0.34 | 0.74 | -0.19 | 0.14 | 0.02 | 0.10 | 0.21 | 0.84 | -0.17 | 0.21 |
| Child IQ | 0.11 | 0.06 | 1.91 | 0.06 | 0.00 | 0.22 | 0.09 | 0.06 | 1.66 | 0.10 | -0.02 | 0.20 |
| Parental attachment | 0.01 | 0.06 | 0.19 | 0.85 | -0.10 | 0.12 | 0.01 | 0.06 | 0.23 | 0.82 | -0.10 | 0.12 |
| Sensitivity (b1) | -0.02 | 0.06 | -0.35 | 0.73 | -0.14 | 0.10 | 0.04 | 0.05 | 0.81 | 0.42 | -0.06 | 0.15 |
| Parent age | 0.02 | 0.01 | 1.79 | 0.07 | 0.00 | 0.04 | 0.01 | 0.01 | 1.29 | 0.20 | -0.01 | 0.04 |
| Child Sex | **0.39** | **0.11** | **3.53** | **0.00** | **0.17** | **0.61** | **0.46** | **0.11** | **4.18** | **0.00** | **0.24** | **0.67** |
| SES | 0.08 | 0.09 | 0.90 | 0.37 | -0.10 | 0.27 | 0.07 | 0.09 | 0.80 | 0.42 | -0.11 | 0.25 |
| **Sensitivity** | **R² = 7.7%, Cohen’s d = 0.58** | | | |  |  | **R² = 3.1%, Cohen’s d = 0.36** | | | |  |  |
| Parent PGS-EDINQ (a1) | -0.02 | 0.10 | -0.21 | 0.84 | -0.21 | 0.17 | 0.06 | 0.11 | 0.53 | 0.60 | -0.16 | 0.28 |
| Parental attachment (a2) | 0.09 | 0.05 | 1.60 | 0.11 | -0.02 | 0.19 | 0.09 | 0.06 | 1.48 | 0.14 | -0.03 | 0.20 |
| SES | **0.37** | **0.09** | **4.38** | **0.00** | **0.21** | **0.54** | **0.19** | **0.09** | **2.06** | **0.04** | **0.01** | **0.37** |
| Intervention group | 0.12 | 0.11 | 1.15 | 0.25 | -0.09 | 0.34 | 0.15 | 0.12 | 1.25 | 0.21 | -0.08 | 0.38 |
| **Parental Attachment** | **R² = 4.2%, Cohen’s d = 0.42** | | | |  |  | **R² = 4.2%, Cohen’s d = 0.42** | | | |  |  |
| Parent PGS-EDINQ | 0.05 | 0.10 | 0.46 | 0.65 | -0.15 | 0.24 | 0.11 | 0.11 | 1.00 | 0.32 | -0.11 | 0.33 |
| Parent sex | 0.36 | 0.20 | 1.80 | 0.07 | -0.03 | 0.76 | 0.37 | 0.20 | 1.82 | 0.07 | -0.03 | 0.77 |
| SES | **0.26** | **0.09** | **3.01** | **0.00** | **0.09** | **0.44** | **0.24** | **0.09** | **2.72** | **0.01** | **0.07** | **0.41** |
| Intervention group | 0.15 | 0.11 | 1.28 | 0.20 | -0.08 | 0.37 | 0.13 | 0.11 | 1.17 | 0.24 | -0.09 | 0.36 |
| **Child PGS-EDINQ** | **R² = 20.6%, Cohen’s d = 1.02** | | | | | | **R² = 16.8%, Cohen’s d = 0.90** | | | |  |  |
| Parent PGS-EDINQ | **0.58** | **0.13** | **4.42** | **0.00** | **0.32** | **0.83** | **0.47** | **0.12** | **3.92** | **0.00** | **0.23** | **0.70** |
| **Mediation** |  |  |  |  |  |  |  |  |  |  |  |  |
| a1*b1 | 0.00 | 0.00 | 0.19 | 0.85 | -0.01 | 0.01 | 0.00 | 0.01 | 0.46 | 0.64 | -0.01 | 0.01 |
| a2*b1 | 0.00 | 0.01 | -0.40 | 0.69 | -0.01 | 0.01 | 0.00 | 0.01 | 0.58 | 0.56 | -0.01 | 0.02 |

*Note.* b = unstandardized parameter estimate, se = standard error, z = Z-statistic, CI = confidence interval; Model fit: Child 1: Χ² (90) = 435.08, p = <.01, CFI = 0.91, TLI = 0.88, RMSEA = .031, Child 2: Χ² (90) = 3772.54, p = <.001, CFI = 0.90, TLI = 0.87, RMSEA = .029; Model fit was poor, which can be expected in models with many variables and a modest sample size, and we therefore did not try to improve model fit using modification indices (Kenny & McCoach, 2003);

| **Table S5** |  |  |  |  |  |  |  |  |  |  |  |  |
| --- | --- | --- | --- | --- | --- | --- | --- | --- | --- | --- | --- | --- |
|  |  |  |  |  |  |  |  |  |  |  |  |  |
| *Structural equation model of parental and child PGS IQ, parental sensitivity, and phenotypic IQ on the child attachment representations using complete data* | | | | | | | | | | | | |
|  |  |  |  |  |  |  |  |  |  |  |  |  |
|  | Child 1 | | | | | | Child 2 | | | | | |
| Predictors | b | se | z | p | CI | | b | se | z | p | CI | |
| **Child Attachment** | **R² = 11.7, Cohen’s d = 0.73** | | | |  |  | **R² = 13.1%, Cohen’s d = 0.78** | | | |  |  |
| Parent PGS-IQ | -0.09 | 0.08 | -1.06 | 0.29 | -0.26 | 0.08 | 0.00 | 0.07 | 0.00 | 1.00 | -0.13 | 0.13 |
| Child PGS-IQ | 0.02 | 0.08 | 0.29 | 0.77 | -0.13 | 0.17 | -0.02 | 0.07 | -0.27 | 0.79 | -0.16 | 0.12 |
| Child IQ | **0.18** | **0.07** | **2.52** | **0.01** | **0.04** | **0.32** | **0.16** | **0.07** | **2.30** | **0.02** | **0.02** | **0.29** |
| Parental attachment | 0.00 | 0.06 | -0.05 | 0.96 | -0.13 | 0.12 | 0.02 | 0.05 | 0.51 | 0.61 | -0.07 | 0.12 |
| Sensitivity (b1) | -0.13 | 0.08 | -1.65 | 0.10 | -0.28 | 0.02 | 0.11 | 0.06 | 1.70 | 0.09 | -0.02 | 0.23 |
| Parent age | **0.03** | **0.01** | **1.78** | **0.08** | **0.00** | **0.05** | 0.03 | 0.01 | 1.97 | 0.05 | 0.00 | 0.06 |
| Child Sex | **0.41** | **0.15** | **2.82** | **0.01** | **0.13** | **0.70** | **0.40** | **0.13** | **3.10** | **0.00** | **0.15** | **0.66** |
| SES | 0.06 | 0.13 | 0.46 | 0.65 | -0.19 | 0.30 | 0.08 | 0.12 | 0.68 | 0.50 | -0.15 | 0.31 |
| **Sensitivity** | **R² = 5.8%, Cohen’s d = 0.50** | | | |  |  | **R² = 4.4%, Cohen’s d = 0.43** | | | |  |  |
| Parent PGS-IQ (a1) | -0.04 | 0.09 | -0.48 | 0.63 | -0.22 | 0.13 | 0.12 | 0.07 | 1.62 | 0.11 | -0.03 | 0.26 |
| Child PGS-IQ (a2) | 0.10 | 0.09 | 1.03 | 0.30 | -0.09 | 0.28 | 0.03 | 0.09 | 0.36 | 0.72 | -0.14 | 0.20 |
| Parental attachment (a3) | 0.11 | 0.07 | 1.57 | 0.12 | -0.03 | 0.24 | 0.04 | 0.07 | 0.56 | 0.58 | -0.10 | 0.17 |
| SES | **0.30** | **0.12** | **2.45** | **0.01** | **0.06** | **0.53** | 0.21 | 0.12 | 1.70 | 0.09 | -0.03 | 0.45 |
| Intervention group | 0.14 | 0.14 | 0.98 | 0.33 | -0.14 | 0.41 | 0.08 | 0.15 | 0.57 | 0.57 | -0.20 | 0.37 |
| **Parental Attachment** | **R² = 4.8%, Cohen’s d = 0.45** | | | |  |  | **R² = 5.6%, Cohen’s d = 0.49** | | | |  |  |
| Parent PGS-IQ | 0.04 | 0.07 | 0.54 | 0.59 | -0.09 | 0.17 | 0.08 | 0.08 | 1.02 | 0.31 | -0.07 | 0.23 |
| Parent sex | **0.57** | **0.19** | **3.01** | **0.00** | **0.20** | **0.95** | **0.48** | **0.19** | **2.53** | **0.01** | **0.11** | **0.85** |
| SES | **0.28** | **0.13** | **2.07** | **0.04** | **0.02** | **0.54** | **0.30** | **0.13** | **2.22** | **0.03** | **0.03** | **0.56** |
| Intervention group | 0.14 | 0.15 | 0.90 | 0.37 | -0.16 | 0.43 | 0.21 | 0.16 | 1.35 | 0.18 | -0.10 | 0.51 |
| **Child PGS-IQ** | **R² = 17.2%, Cohen’s d = 0.91** | | | |  |  | **R² = 11.6%, Cohen’s d = 0.72** | | | |  |  |
| Parent PGS-IQ | **0.44** | **0.07** | **6.26** | **0.00** | **0.30** | **0.58** | **0.33** | **0.07** | **4.82** | **0.00** | **0.20** | **0.47** |
| **Mediation** |  |  |  |  |  |  |  |  |  |  |  |  |
| a1*b1 | 0.01 | 0.01 | 0.47 | 0.64 | -0.02 | 0.03 | 0.01 | 0.01 | 1.20 | 0.23 | -0.01 | 0.03 |
| a2*b1 | -0.01 | 0.02 | -0.84 | 0.40 | -0.04 | 0.02 | 0.00 | 0.01 | 0.35 | 0.73 | -0.02 | 0.02 |
| a3*b1 | -0.01 | 0.01 | -1.20 | 0.23 | -0.04 | 0.01 | 0.00 | 0.01 | 0.54 | 0.59 | -0.01 | 0.02 |
| *Note.* b = unstandardized parameter estimate, se = standard error, z = Z-statistic, CI = confidence interval; Model fit: Child 1: Χ² (34) = 96.96, p <.001, CFI = 0.93, TLI = 0.86, RMSEA = .036, Child 2: Χ² (34) = 94.60, p = .001, CFI = 0.88 TLI = 0.74, RMSEA = .048; Model fit was poor, which can be expected in models with many variables and a modest sample size, and we therefore did not try to improve model fit using modification indices (Kenny & McCoach, 2003); | | | | | | | | | | | | |

| **Table S6** |  |  |  |  |  |  |  |  |  |  |  |  |
| --- | --- | --- | --- | --- | --- | --- | --- | --- | --- | --- | --- | --- |
|  |  |  |  |  |  |  |  |  |  |  |  |  |
| *Structural equation model of parental and child PGS IQ, parental sensitivity, and phenotypic IQ on the child attachment representations excluding the prediction of parental attachment by parental (PGS) IQ* | | | | | | | | | | | | |
|  |  |  |  |  |  |  |  |  |  |  |  |  |
|  | Child 1 | | | | | | Child 2 | | | | | |
| Predictors | b | se | z | p | CI | | b | se | z | p | CI | |
| **Child Attachment** | **R² = 6.4%, Cohen’s d = 0.52** | | | |  |  | **R² = 7.7%, Cohen’s d = 0.58** | | | |  |  |
| Parent PGS-IQ | -0.03 | 0.06 | -0.52 | 0.61 | -0.15 | 0.09 | 0.03 | 0.06 | 0.49 | 0.63 | -0.09 | 0.14 |
| Child PGS-IQ | 0.02 | 0.06 | 0.33 | 0.75 | -0.09 | 0.13 | 0.02 | 0.06 | 0.43 | 0.67 | -0.09 | 0.14 |
| Child IQ | 0.10 | 0.06 | 1.89 | 0.06 | 0.00 | 0.21 | 0.09 | 0.05 | 1.67 | 0.10 | -0.02 | 0.20 |
| Parental attachment | 0.01 | 0.06 | 0.18 | 0.86 | -0.10 | 0.12 | 0.01 | 0.05 | 0.22 | 0.83 | -0.09 | 0.12 |
| Sensitivity (b1) | -0.02 | 0.06 | -0.37 | 0.71 | -0.14 | 0.09 | 0.04 | 0.05 | 0.82 | 0.42 | -0.06 | 0.14 |
| Parent age | 0.02 | 0.01 | 1.76 | 0.08 | 0.00 | 0.04 | 0.01 | 0.01 | 1.21 | 0.23 | -0.01 | 0.03 |
| Child Sex | **0.39** | **0.11** | **3.51** | **0.00** | **0.17** | **0.60** | **0.46** | **0.11** | **4.36** | **0.00** | **0.25** | **0.66** |
| SES | 0.07 | 0.09 | 0.70 | 0.49 | -0.12 | 0.25 | 0.07 | 0.09 | 0.76 | 0.45 | -0.11 | 0.24 |
| **Sensitivity** | **R² = 7.6%, Cohen’s d = 0.57** | | | |  |  | **R² = 3.6%, Cohen’s d = 0.39** | | | |  |  |
| Parent PGS-IQ (a1) | -0.02 | 0.06 | -0.30 | 0.77 | -0.13 | 0.09 | 0.04 | 0.06 | 0.75 | 0.46 | -0.07 | 0.16 |
| Parental attachment (a2) | 0.09 | 0.05 | 1.63 | 0.10 | -0.02 | 0.19 | 0.09 | 0.06 | 1.57 | 0.12 | -0.02 | 0.20 |
| Child Age | **0.37** | **0.09** | **4.34** | **0.00** | **0.20** | **0.54** | **0.19** | **0.09** | **2.12** | **0.03** | **0.02** | **0.37** |
| Intervention group | 0.12 | 0.11 | 1.16 | 0.25 | -0.09 | 0.33 | 0.14 | 0.11 | 1.27 | 0.21 | -0.08 | 0.37 |
| **Child PGS-IQ** | **R² = 5.6%, Cohen’s d = 0.49** | | | |  |  | **R² = 3.4%, Cohen’s d = 0.38** | | | |  |  |
| Parent PGS-IQ | **0.25** | **0.06** | **4.32** | **0.00** | **0.14** | **0.37** | **0.18** | **0.05** | **3.50** | **0.00** | **0.08** | **0.29** |
| **Mediation** |  |  |  |  |  |  |  |  |  |  |  |  |
| a1*b1 | 0.00 | 0.00 | 0.24 | 0.81 | 0.00 | 0.00 | 0.00 | 0.00 | 0.44 | 0.66 | -0.01 | 0.01 |
| a2*b1 | 0.00 | 0.01 | -0.39 | 0.70 | -0.01 | 0.01 | 0.00 | 0.01 | 0.58 | 0.56 | -0.01 | 0.02 |
| *Note.* b = unstandardized parameter estimate, se = standard error, z = Z-statistic, CI = confidence interval; Model fit: Child 1: Χ² (24) = 62.00, p < .001, CFI = 0.95, TLI = 0.90, RMSEA = .018, Child 2: Χ² (24) = 48.41, p = .002, CFI = 0.88, TLI = 0.73, RMSEA = .024; Model fit was poor, which can be expected in models with many variables and a modest sample size, and we therefore did not try to improve model fit using modification indices (Kenny & McCoach, 2003); | | | | | | | | | | | | |

| **Table S7** |  |  |  |  |  |  |  |  |  |  |  |  |
| --- | --- | --- | --- | --- | --- | --- | --- | --- | --- | --- | --- | --- |
|  |  |  |  |  |  |  |  |  |  |  |  |  |
| *Structural equation model of parental and child PGS IQ, parental sensitivity, and phenotypic IQ on the child attachment representations using child sex and age as covariate and excluding SES* | | | | | | | | | | | | |
|  |  |  |  |  |  |  |  |  |  |  |  |  |
|  | Child 1 | | | | | | Child 2 | | | | | |
| Predictors | b | se | z | p | CI | | b | se | z | p | CI | |
| **Child Attachment** | **R² = 8.9, Cohen’s d = 0.63** | | |  |  |  | **R² = 10.9, Cohen’s d = 0.70** | | |  |  |  |
| Parent PGS-IQ | 0.00 | 0.06 | -0.05 | 0.96 | -0.12 | 0.11 | 0.04 | 0.06 | 0.63 | 0.53 | -0.08 | 0.15 |
| Child PGS-IQ | 0.03 | 0.05 | 0.57 | 0.57 | -0.07 | 0.13 | 0.06 | 0.06 | 1.04 | 0.30 | -0.05 | 0.17 |
| Child IQ | 0.10 | 0.05 | 1.99 | 0.05 | 0.00 | 0.20 | 0.07 | 0.05 | 1.38 | 0.17 | -0.03 | 0.18 |
| Parental attachment | 0.01 | 0.05 | 0.16 | 0.88 | -0.10 | 0.11 | 0.02 | 0.05 | 0.31 | 0.76 | -0.09 | 0.12 |
| Sensitivity (b1) | 0.00 | 0.05 | 0.06 | 0.95 | -0.10 | 0.11 | 0.08 | 0.05 | 1.50 | 0.14 | -0.02 | 0.18 |
| Parent age | 0.00 | 0.01 | 0.23 | 0.82 | -0.02 | 0.03 | 0.00 | 0.01 | -0.39 | 0.70 | -0.03 | 0.02 |
| Child Sex | **0.39** | **0.11** | **3.73** | **0.00** | **0.19** | **0.60** | **0.44** | **0.10** | **4.28** | **0.00** | **0.24** | **0.64** |
| Child Age | **0.08** | **0.03** | **2.98** | **0.00** | **0.03** | **0.13** | **0.09** | **0.03** | **3.60** | **0.00** | **0.04** | **0.15** |
| **Sensitivity** | **R² = 3.0, Cohen’s d = 0.35** | | |  |  |  | **R² = 3.8, Cohen’s d = 0.40** | | |  |  |  |
| Parent PGS-IQ (a1) | 0.00 | 0.06 | -0.01 | 0.99 | -0.11 | 0.11 | 0.06 | 0.06 | 1.10 | 0.27 | -0.05 | 0.18 |
| Child PGS-IQ (a2) | 0.07 | 0.05 | 1.32 | 0.19 | -0.03 | 0.17 | 0.01 | 0.06 | 0.20 | 0.84 | -0.10 | 0.13 |
| Parental attachment (a3) | **0.12** | **0.05** | **2.32** | **0.02** | **0.02** | **0.22** | **0.12** | **0.06** | **2.12** | **0.03** | **0.01** | **0.22** |
| Child Sex | -0.06 | 0.10 | -0.60 | 0.55 | -0.26 | 0.14 | 0.05 | 0.11 | 0.48 | 0.64 | -0.16 | 0.27 |
| Child Age | -0.03 | 0.02 | -1.23 | 0.22 | -0.07 | 0.02 | **-0.06** | **0.02** | **-2.32** | **0.02** | **-0.11** | **-0.01** |
| Intervention group | 0.14 | 0.11 | 1.28 | 0.20 | -0.07 | 0.34 | 0.16 | 0.11 | 1.45 | 0.15 | -0.06 | 0.39 |
| **Parental Attachment** | **R² = 1.6, Cohen’s d = 0.26** | | |  |  |  | **R² = 2.1, Cohen’s d = 0.29** | | |  |  |  |
| Parent PGS-IQ | 0.05 | 0.06 | 0.80 | 0.43 | -0.07 | 0.16 | 0.08 | 0.06 | 1.45 | 0.15 | -0.03 | 0.19 |
| Parent sex | 0.30 | 0.19 | 1.55 | 0.12 | -0.08 | 0.68 | 0.32 | 0.20 | 1.63 | 0.10 | -0.06 | 0.70 |
| Child Sex | -0.06 | 0.11 | -0.57 | 0.57 | -0.27 | 0.15 | -0.06 | 0.11 | -0.59 | 0.55 | -0.28 | 0.15 |
| Child Age | 0.02 | 0.02 | 0.64 | 0.52 | -0.03 | 0.06 | 0.01 | 0.02 | 0.48 | 0.63 | -0.04 | 0.06 |
| Intervention group | 0.13 | 0.11 | 1.21 | 0.23 | -0.08 | 0.35 | 0.13 | 0.11 | 1.19 | 0.24 | -0.09 | 0.35 |
| **Child PGS-IQ** | **R² = 5.1, Cohen’s d = 0.46** | | |  |  |  | **R² = 3.1, Cohen’s d = 0.36** | | |  |  |  |
| Parent PGS-IQ | **0.24** | **0.06** | **4.29** | **0.00** | **0.13** | **0.35** | **0.19** | **0.05** | **3.65** | **0.00** | **0.09** | **0.30** |
| **Mediation** |  | |  |  |  |  |  |  |  |  |  |  |
| a1*b1 | 0.00 | 0.00 | -0.05 | 0.96 | 0.00 | 0.00 | 0.01 | 0.01 | 0.80 | 0.43 | -0.01 | 0.02 |
| a2*b1 | 0.00 | 0.00 | 0.09 | 0.93 | -0.01 | 0.01 | 0.00 | 0.01 | 0.16 | 0.87 | -0.01 | 0.01 |
| a3*b1 | 0.00 | 0.01 | 0.05 | 0.96 | -0.01 | 0.01 | 0.01 | 0.01 | 1.35 | 0.18 | 0.00 | 0.02 |
| *Note.* b = unstandardized parameter estimate, se = standard error, z = Z-statistic, CI = confidence interval; Model fit: Child 1: Χ² (34) = 67.97, p <.001, CFI = 0.95, TLI = 0.88, RMSEA = .016, Child 2: Χ² (34) = 86.08, p < .001, CFI = 0.76 TLI = 0.42, RMSEA = .042; Model fit was poor, which can be expected in models with many variables and a modest sample size, and we therefore did not try to improve model fit using modification indices (Kenny & McCoach, 2003); | | | | | | | | | | | | |

| **Table S8** |  |  |  |  |  |  |  |  |  |  |  |  |
| --- | --- | --- | --- | --- | --- | --- | --- | --- | --- | --- | --- | --- |
|  |  |  |  |  |  |  |  |  |  |  |  |  |
| *Structural equation model of parental and child PGS IQ, parental sensitivity, and phenotypic IQ on the child attachment representations using PGS with p-value threshold = 1* | | | | | | | | | | | | |
|  |  |  |  |  |  |  |  |  |  |  |  |  |
|  | Child 1 | | | | | | Child 2 | | | | | |
| Predictors | b | se | z | p | CI | | b | se | z | p | CI | |
| **Child Attachment** | **R² = 6.8, Cohen’s d = 0.54** | | | |  |  | **R² = 7.5%, Cohen’s d = 0.57** | | | |  |  |
| Parent PGS-IQ | -0.02 | 0.06 | -0.42 | 0.68 | -0.14 | 0.09 | 0.02 | 0.06 | 0.30 | 0.77 | -0.10 | 0.13 |
| Child PGS-IQ | 0.01 | 0.05 | 0.26 | 0.79 | -0.09 | 0.12 | 0.01 | 0.06 | 0.15 | 0.88 | -0.10 | 0.12 |
| Child IQ | 0.10 | 0.05 | 1.90 | 0.06 | 0.00 | 0.20 | 0.08 | 0.05 | 1.46 | 0.15 | -0.03 | 0.19 |
| Parental attachment | 0.01 | 0.05 | 0.14 | 0.89 | -0.10 | 0.11 | 0.02 | 0.05 | 0.29 | 0.77 | -0.09 | 0.12 |
| Sensitivity (b1) | -0.02 | 0.06 | -0.29 | 0.77 | -0.13 | 0.09 | 0.05 | 0.05 | 1.00 | 0.32 | -0.05 | 0.15 |
| Parent age | 0.02 | 0.01 | 1.70 | 0.09 | 0.00 | 0.04 | 0.02 | 0.01 | 1.41 | 0.16 | -0.01 | 0.04 |
| Child Sex | **0.40** | **0.11** | **3.78** | **0.00** | **0.19** | **0.60** | **0.45** | **0.11** | **4.32** | **0.00** | **0.25** | **0.66** |
| SES | 0.08 | 0.09 | 0.90 | 0.37 | -0.10 | 0.26 | 0.08 | 0.09 | 0.90 | 0.37 | -0.09 | 0.25 |
| **Sensitivity** | **R² = 7.1, Cohen’s d = 0.55** | | | |  |  | **R² = 3.6%, Cohen’s d = 0.39** | | | |  |  |
| Parent PGS-IQ (a1) | -0.03 | 0.06 | -0.51 | 0.61 | -0.14 | 0.08 | 0.04 | 0.06 | 0.68 | 0.50 | -0.08 | 0.16 |
| Child PGS-IQ (a2) | 0.04 | 0.05 | 0.71 | 0.48 | -0.06 | 0.13 | 0.01 | 0.06 | 0.17 | 0.86 | -0.11 | 0.13 |
| Parental attachment (a3) | 0.08 | 0.05 | 1.66 | 0.10 | -0.02 | 0.18 | 0.09 | 0.06 | 1.67 | 0.10 | -0.02 | 0.20 |
| SES | **0.35** | **0.08** | **4.37** | **0.00** | **0.20** | **0.51** | **0.20** | **0.09** | **2.20** | **0.03** | **0.02** | **0.37** |
| Intervention group | 0.12 | 0.10 | 1.19 | 0.23 | -0.08 | 0.32 | 0.15 | 0.11 | 1.29 | 0.20 | -0.08 | 0.37 |
| **Parental Attachment** | **R² = 4.2, Cohen’s d = 0.42** | | | |  |  | **R² = 4.4%, Cohen’s d = 0.43** | | | |  |  |
| Parent PGS-IQ | 0.01 | 0.06 | 0.25 | 0.80 | -0.10 | 0.12 | 0.05 | 0.06 | 0.79 | 0.43 | -0.07 | 0.16 |
| Parent sex | 0.36 | 0.19 | 1.89 | 0.06 | -0.01 | 0.73 | 0.37 | 0.19 | 1.91 | 0.06 | -0.01 | 0.75 |
| SES | **0.27** | **0.08** | **3.21** | **0.00** | **0.11** | **0.44** | **0.26** | **0.09** | **3.02** | **0.00** | **0.09** | **0.43** |
| Intervention group | 0.13 | 0.11 | 1.24 | 0.22 | -0.08 | 0.34 | 0.13 | 0.11 | 1.23 | 0.22 | -0.08 | 0.35 |
| **Child PGS-IQ** | **R² = 5.1, Cohen’s d = 0.46** | | | |  |  | **R² = 3.1%, Cohen’s d = 0.36** | | | |  |  |
| Parent PGS-IQ | **0.24** | **0.06** | **4.33** | **0.00** | **0.13** | **0.35** | **0.19** | **0.05** | **3.64** | **0.00** | **0.09** | **0.30** |
| **Mediation** |  |  |  |  |  |  |  |  |  |  |  |  |
| a1*b1 | 0.00 | 0.00 | 0.11 | 0.91 | -0.01 | 0.01 | 0.00 | 0.00 | 0.44 | 0.66 | -0.01 | 0.01 |
| a2*b1 | 0.00 | 0.00 | -0.39 | 0.69 | 0.00 | 0.00 | 0.00 | 0.00 | 0.11 | 0.91 | -0.01 | 0.01 |
| a3*b1 | 0.00 | 0.01 | -0.31 | 0.76 | -0.01 | 0.01 | 0.01 | 0.00 | 1.13 | 0.26 | 0.00 | 0.01 |
| *Note.* b = unstandardized parameter estimate, se = standard error, z = Z-statistic, CI = confidence interval; Model fit: Child 1: Χ² (34) = 68.16, p <.001, CFI = 0.96, TLI = 0.92, RMSEA = .015, Child 2: Χ² (34) = 78.62, p = .001, CFI = 0.94 TLI = 0.87, RMSEA = .019; Model fit was poor, which can be expected in models with many variables and a modest sample size, and we therefore did not try to improve model fit using modification indices (Kenny & McCoach, 2003); | | | | | | | | | | | | |
